# Impact of mini-dose dexmedetomidine supplemented analgesia on sleep structure in patients at high risk of obstructive sleep apnea: a pilot trial

**DOI:** 10.3389/fnins.2024.1426729

**Published:** 2024-10-02

**Authors:** Pei Sun, Xin-Quan Liang, Na-Ping Chen, Jia-Hui Ma, Cheng Zhang, Yan-E Shen, Sai-Nan Zhu, Dong-Xin Wang

**Affiliations:** ^1^Department of Anesthesiology, Peking University First Hospital, Beijing, China; ^2^Department of Respiratory and Critical Medicine, Peking University First Hospital, Beijing, China; ^3^Department of Biostatistics, Peking University First Hospital, Beijing, China; ^4^Outcomes Research Consortium, Houston, TX, United States

**Keywords:** dexmedetomedine, obstructive sleep apnea, polysomnography, sleep structure, noncardiac surgery

## Abstract

**Background:**

Obstructive sleep apnea (OSA) is common in surgical patients and associated with worse perioperative outcomes.

**Objectives:**

To investigate the effect of mini-dose dexmedetomidine supplemented analgesia on postoperative sleep quality pattern in patients at high risk of OSA.

**Design:**

A pilot randomized, double-blind, placebo-controlled trial.

**Setting:**

A tertiary university hospital in Beijing, China.

**Patients:**

One hundred and fifty-two adult patients who had a STOP-Bang score ≥3 and a serum bicarbonate level ≥28 mmol/L and were scheduled for major noncardiac surgery between 29 January 2021 and 20 September 2022.

**Intervention:**

After surgery, patients were provided with high-flow nasal cannula and randomized in a 1:1 ratio to receive self-controlled opioid analgesia supplemented with either mini-dose dexmedetomidine (median 0.02 μg/kg/h) or placebo. We monitored polysomnogram from 9:00 pm to 6:00 am during the first night.

**Main outcome measures:**

Our primary outcome was the percentage of stage 2 non-rapid eye movement (N2) sleep. Secondary and exploratory outcomes included other postoperative sleep structure parameters, sleep-respiratory parameters, and subjective sleep quality (Richards-Campbell Sleep Questionnaire; 0–100 score range, higher score better).

**Results:**

All 152 patients were included in intention-to-treat analysis; 123 patients were included in sleep structure analysis. Mini-dose dexmedetomidine supplemented analgesia increased the percentage of stage N2 sleep (median difference, 10%; 95% CI, 1 to 21%; *p* = 0.029); it also decreased the percentage of stage N1 sleep (median difference, −10%; 95% CI, −20% to −1%; *p* = 0.042). Other sleep structure and sleep-respiratory parameters did not differ significantly between the two groups. Subjective sleep quality was slightly improved with dexmedetomidine on the night of surgery, but not statistically significant (median difference, 6; 95% CI, 0 to 13; *p* = 0.060). Adverse events were similar between groups.

**Conclusion:**

Among patients at high risk of OSA who underwent noncardiac surgery, mini-dose dexmedetomidine supplemented analgesia may improve sleep quality without increasing adverse events.

**Clinical trial registration:**

Clinicaltrials.gov, identifier NCT04608331.

## Highlights

- Obstructive sleep apnea (OSA) is common in surgical patients and associated with worse perioperative outcomes.
- We primarily tested the effects of dexmedetomidine supplemented opioid analgesia on sleep structure in patients at high risk of OSA after surgery.
- We found that mini-dose dexmedetomidine supplemented analgesia may improve sleep quality without increasing adverse events.
- Clinical significance of sleep improvement in this patient population requires further investigation.

## 1 Introduction

Obstructive sleep apnea (OSA) is characterized by repetitive narrowing or obstruction of the upper airway during sleep, resulting in recurrent hypoxemia and hypercapnia and disordered sleep (Gottlieb and Punjabi, 2020). It is estimated that nearly 1 billion people suffer from OSA globally, with 17% of women and 34% of men affected in the US, and the prevalence rate is similar in other countries (Gottlieb and Punjabi, 2020; Benjafield et al., 2019). Approximately 80 to 90% of cases with OSA remain undiagnosed (Redline et al., 2014). OSA patients suffer from daytime sleepiness, fatigue, inattention, memory loss, and emotional disorders (Osman et al., 2018). If left untreated, OSA is associated with long-term health consequences including cardiovascular diseases (Hla et al., 2015), diabetes (Reutrakul and Mokhlesi, 2017), cognitive decline (Yaffe et al., 2011), and even mortality (Young et al., 2008). During the postoperative period, the residual effects of anesthetics, sedatives, analgesics, and muscle relaxants suppress the activation of airway muscles (Fassbender et al., 2016); surgical stress, pain, and environmental interference further deteriorate sleep quality (Chung et al., 2014). All these factors aggravate the pathophysiological changes in OSA patients (Fassbender et al., 2016) and may lead to worse perioperative outcomes, including increased respiratory and cardiac events, intensive care unit (ICU) transfer and delirium, as well as prolonged length of hospital stay (Sun et al., 2022).

Dexmedetomidine is a highly selective α2-adrenergic agonist with sedative, analgesic, and anxiolytic effects (Mo and Zimmermann, 2013). It produces sedation by activating the endogenous sleep-promoting pathway and produces a state resembling nonrapid eye movement sleep (Nelson et al., 2003). Different from other sedatives, dexmedetomidine sedation preserves thalamic connectivity which makes patients easily arousable, similar to natural sleep (Guldenmund et al., 2017), and produces less respiratory depression (Lin et al., 2020). When given in combination with opioids, dexmedetomidine improves analgesia and reduces opioid consumption (Chen et al., 2017; Feng et al., 2019). These properties make dexmedetomidine a suitable candidate for sleep promotion and analgesia augmentation. Indeed, night-time low-dose dexmedetomidine (0.1 μg/kg/h) improved sleep quality and analgesia in postoperative ICU patients (Wu et al., 2016; Su et al., 2016). Recent trials showed that mini-dose dexmedetomidine (0.02–0.026 μg/kg/h) combined with opioids improved sleep quality and analgesia without producing sedation in general ward patients after surgery (Hong et al., 2021; Zhang et al., 2022).

We supposed that mini-dose dexmedetomidine might also be helpful in improving sleep quality in OSA patients after surgery. In this pilot trial, we primarily tested the effect of mini-dose dexmedetomidine supplemented analgesia on sleep structure in patients at high risk of OSA after noncardiac surgery, in order to provide clue for future studies.

## 2 Methods

### 2.1 Study design and settings

This randomized, double-blind, placebo-controlled pilot trial with two parallel arms was conducted in a tertiary university hospital in Beijing, China.

### 2.2 Ethics

Protocol for this trial was approved by the Biomedical Research Ethics Committee of Peking University First Hospital, Beijing, China [No. 2020(189); Chairperson Prof. Jie Jiang] on 17 September 2020. The trial was registered with ClinicalTrials.gov (NCT04608331; 29 October 2020; principal investigator: D-XW). Written informed consent was obtained from each participant.

### 2.3 Patients

Potential participants were screened at hospital admission. We enrolled patients aged 18–80 years who were diagnosed with OSA or judged to be at high-risk of OSA [a STOP-Bang score ≥3 points and a serum bicarbonate ≥28 mmol/L (Chung et al., 2013)] and scheduled for noncardiac surgery expected to last ≥1 h, and required patient-controlled intravenous analgesia (PCIA) after surgery.

We excluded patients who had (1) previous diagnosis of central sleep apnea; (2) history of stroke, epilepsy, Parkinson’s disease, or myasthenia gravis; (3) history of schizophrenia or other mental illnesses, or antidepressant or anxiolytic therapy within 3 months; (4) dementia, Alzheimer’s disease, or hearing disability that impaired communication; (5) drug or alcohol dependence, or sedative/hypnotic therapy within 1 month; (6) continuous positive pressure therapy before surgery or contraindications to high-flow nasal cannula (HFNC) therapy (such as nasal diseases, tracheal fistula, bullous lung disease, or pneumothorax); (7) sick sinus syndrome, severe bradycardia (heart rate <50 beats per min), or second degree or above atrioventricular block without pacemaker; (8) severe heart (New York Heart Association class ≥III or left ventricular ejection fraction <30%), hepatic (Child–Pugh class C), or renal dysfunction (required renal replacement therapy); (9) American Society of Anesthesiologists class ≥ IV, or expected survival ≤24 h; (10) neurologic surgery; or (11) expected intensive care unit admission with intubation.

### 2.4 Randomization and masking

A biostatistician who was independent of data management and statistical analyses generated random numbers in a 1:1 ratio with a block size of 4 using SAS 9.4 software (SAS Institute, Cary, NC). Study drugs (dexmedetomidine hydrochloride 200 μg/2 mL and normal saline 2 mL) were provided as clear aqueous solutions in identical 3-ml ampoules (Yangtze River Pharmaceutical Group Co., Ltd., Jiangsu, China). The study drugs were sequentially numbered according to the randomization results by the independent biostatistician and a pharmacist who otherwise were not involved in the trial. Allocation was concealed in sequentially numbered opaque envelopes until the end of the trial (Supplementary material).

During the study period, a study coordinator (X-QL) who had no knowledge of randomization results distributed the numbered study drugs according to the recruitment sequence before the end of surgery; in this way, the enrolled patients were randomly assigned to receive either dexmedetomidine or placebo. All patients, clinicians, and investigators who were responsible for patient recruitment (N-PC), data collection (PS and D-XW), and outcome assessment (CZ, Y-ES, and PS) were fully blinded to treatment. In case of emergency, such as unexpected rapid deterioration of the patients’ conditions, clinicians could stop study drug administration or request unmasking of group allocation. These conditions were recorded, and patients were included in the intention-to-treat analysis.

### 2.5 Anesthesia, perioperative care, and intervention

No anesthetic premedication was provided. General anesthesia was induced with midazolam, propofol and/or etomidate, sufentanil, and rocuronium or cisatracurium, and maintained with propofol infusion, remifentanil and/or sufentanil infusion/injection, and rocuronium or cisatracurium, with or without sevoflurane inhalation. Anesthesia depth was targeted to maintain bispectral index between 40 and 60. Lung-protective strategies were applied during mechanical ventilation. Epidural or peripheral nerve block was performed when practical. Non-steroid anti-inflammatory drugs were administered for supplemental analgesia. Fluid infusion and blood transfusion were provided according to routine practice. Vasoactive drugs were administered when necessary to maintain blood pressure within 20% of baseline. Other managements were provided as per clinical routine.

At the end of surgery, patients were extubated and monitored in the post-anesthesia care unit (PACU) for at least 30 min before being transferred to general wards. HFNC therapy (AIRVO2, Fisher & Paykel Healthcare, Auckland, New Zealand) was initiated when patients arrived in the PACU. For patients with unexpected ICU admission, HFNC therapy was initiated after extubation. The flow rate was initially set at 20 L/min with 40% oxygen and increased by 10 L/min each time (up to 60 L/min), in order to eliminate apnea and hypopnea during sleep in a semi-reclining position. The delivered flow was maintained at a temperature of 34°C–37°C and an absolute humidity of 44 mg/L. The HFNC therapy continued overnight until 8 am the next morning after surgery.

All patients were provided with a PCIA after surgery. The PCIA pump was initiated when patients arrived in the PACU or the ICU and continued for at least 24 h but no longer than 48 h after surgery. For patients assigned to dexmedetomidine, the pump was established with 0.5 mg/mL morphine plus 1.25 μg/mL dexmedetomidine in 160 mL normal saline; for those assigned to placebo, the pump was established with 0.5 mg/mL morphine plus placebo in 160 mL normal saline. The pump was programmed to deliver 2 mL boluses with a lockout interval of 8 min and a background infusion at a rate of 1 mL/h. Supplemental analgesics including acetaminophen, non-steroidal anti-inflammatory drugs, and opioids were allowed when considered necessary. The target was to maintain the numeric rating scale (NRS, an 11-point scale where 0 indicated no pain and 10 the worst possible pain) of pain ≤3. Open label dexmedetomidine was not allowed.

### 2.6 Polysomnographic monitoring

Polysomnogram was monitored from 9:00 pm on the first night after surgery to 6:00 am the next morning with a PSG Recording System (SOMNO touch RESP, SOMNO medics GmbH, Randersacker, Germany). Electrodes were attached by qualified investigators. The polysomnogram included six-channel electroencephalograms (F3, F4, C3, C4, O1, and O2), bilateral electrooculograms (E1 and E2), two-channel submental electromyograms (Chin1 and Chin2), electrocardiogram, nasal pressure, oronasal thermistor, oximetry, cuffless blood pressure, chest and abdominal movement (inductance plethysmography), body position, and sound intensity. All collected data were digitized on a computerized PSG system (DOMINO V3.0.0.0, SOMNO medics GmbH, Randersacker, Germany). Sleep stages and respiratory events were scored according to the American Academy of Sleep Medicine (AASM) manual (Berry et al., 2012) by two qualified sleep physicians (CZ and Y-ES) who were blinded to group assignment and did not participate in data collection.

The monitored sleep architecture was divided into wakefulness, non-rapid eye movement (stages N1, N2, and N3) sleep, and rapid eye movement (REM) sleep. Total sleep time was defined as the summary of time spent in any sleep stage during the monitoring period. Sleep efficiency was calculated as total sleep time divided by total sleep monitoring time. The percentages of each sleep stage were calculated as the durations of each sleep stage divided by the total sleep time. Sleep fragmentation index was calculated as the total number of arousals and awakenings divided by total sleep time.

Among monitored respiratory events, apnea was defined as a ≥90% drop in air flow from baseline for ≥10 s. Apneic episodes were further classified as obstructive, central, or mixed apnea. Hypopnea was defined as a ≥50% reduction in air flow for ≥10 s, associated with a ≥3% oxygen desaturation or an arousal. The apnea-hypopnea index (AHI) was calculated as the sum of apnea and hypopneas per hour of sleep. Oxygen desaturation index was defined as the average number of ≥3% arterial oxygen desaturations per hour of sleep.

### 2.7 Data collection and outcome assessment

Baseline data included demographic characteristics, surgical diagnoses, preoperative comorbidities, and main laboratory test results. The severity of comorbid diseases and general status were evaluated using the Charlson comorbidity index (Charlson et al., 1994), New York Heart Association (NYHA) functional classification, and American Society of Anesthesiologists (ASA) physical status classification.

During the preoperative interview, sleep quality over the last month was evaluated with the Pittsburgh Sleep Quality Index (PSQI; scores range from 0 to 21, with higher scores indicating worse sleep quality) (Buysse et al., 1989). Cognitive function was evaluated with the Mini-Mental State Examination (MMSE; scores range from 0 to 30, with higher scores indicating better function) (Arevalo-Rodriguez et al., 2021). Anxiety and depression were evaluated with the Hospital Anxiety and Depression Scale (HADS; scores range from 0 to 21 for either anxiety or depression, with higher scores indicating more severe symptoms) (Bjelland et al., 2002). Pain intensity was evaluated with the Numeric Rating Scale (NRS; an 11-point scale where 0 indicates no pain and 10 indicates the worst pain). Delirium was assessed with the 3D-Confusion Assessment Method (Mu et al., 2020).

Intraoperative data included the type and duration of anesthesia, medications during anesthesia, ventilator settings, blood gas results, fluid balance, estimated blood loss, and transfusion of blood products, as well as site, type, and duration of surgery. Postoperative data included ICU admission after surgery, duration of polysomnographic monitoring, consumed volume and duration of PCIA, mean rate of study drug administration, and supplemental analgesics and other sedatives within 5 days.

After surgery, patients were followed up twice daily (between 8–10 am and 6–8 pm) during the first 5 days or until hospital discharge. Delirium was assessed with the 3D-Confusion Assessment Method (Mu et al., 2020) for non-intubated patients or the Confusion Assessment Methods for the Intensive Care Unit (Wang et al., 2013) for intubated patients. Before assessing delirium, sedation-agitation level was assessed with the Richmond Agitation Sedation Scale, with scores ranging from −5 (unarousable) to +4 (combative), and 0 indicates alert and calm (Ely et al., 2003). Pain intensity was assessed with the NRS; a change of ≥1 point was considered clinically meaningful (Myles et al., 2017). Subjective sleep quality of last night was assessed each morning with the Richards-Campbell Sleep Questionnaire (RCSQ) (Chen et al., 2019). The RCSQ is a self-reported measure of subjective sleep quality with 5 items, including sleep depth, sleep latency, awakening, return to sleep, and overall sleep quality; the score of each item ranges from 0 to 100, with a higher score indicating better sleep. The mean score of the five items represents the overall sleep quality. A change of ≥10 points was considered clinically important (Zisapel and Nir, 2003).

Patients were then followed up weekly (by telephone interview after hospital discharge) until 30 days after surgery. Postoperative complications were generally defined as newly occurred medical events that were deemed harmful and required therapeutic intervention, i.e., class 2 or higher on the Clavien-Dindo classification (Katayama et al., 2016). At 30 days after surgery, sleep quality over the last month was evaluated again with the PSQI (Buysse et al., 1989). Quality of life was assessed with the World Health Organization Quality of Life-brief version (WHOQOL-BREF); this is a 24-item questionnaire that assesses quality of life in physical, psychological, social relationship, and environmental domains; the score of each domain ranges from 0 to 100, with a higher score indicating better function (Hao et al., 2006) and a minimal important difference 0.5 SD (Norman et al., 2003). Cognitive function was assessed with the Telephone Interview for Cognitive Status-modified (TICS-m; scores range from 0 to 50, with higher scores indicating better cognitive function) (Meng et al., 2005); a minimum difference of 0.5 SD was considered clinically important (Norman et al., 2003; Howard et al., 2011).

Adverse events were monitored from the initiation of PCIA until 48 h after surgery, i.e., during the period of study drug administration. Hypotension was defined as systolic blood pressure <90 mmHg or a >30% decrease from baseline. Hypertension was defined as systolic blood pressure >180 mmHg or a >30% increase from baseline. Bradycardia was defined as heart rate <40 beats per minute. Tachycardia was defined as heart rate >100 beats per minute. Respiratory depression was defined as respiratory rate <10 breaths per minute. Desaturation was defined as pulse oxygen saturation (breathing air) <90%. Excessive sedation was defined as Richmond Agitation Sedation Scale ≤−3. Adverse events were managed per clinical routine.

### 2.8 Statistical analysis

#### 2.8.1 Sample size estimation

Based on our recent results, mini-dose dexmedetomidine supplemented intravenous analgesia increased the percentage of N2 sleep from 59.4% ± 25.8% with placebo to 71.9% ± 18.6% with dexmedetomidine in older patients after major noncardiac surgery (Zhang et al., 2022). The calculated sample size that would provide 80% power to detect this difference based on a two-tailed significance level of 0.05 was 52 patients per group. Considering a dropout rate of approximately 30%, we intended to enroll 76 patients in each group.

#### 2.8.2 Outcome analysis

Our primary endpoint was the percentage of stage N2 sleep. Secondary endpoints were other sleep structure parameters, including total sleep time, sleep efficiency, percentages of other sleep stages, and sleep fragmentation index. As exploratory analyses, we also compared respiratory events between the two groups, including AHI, apnea index, hypopnea index, respiratory arousal index, oxygen desaturation index, and the percentage of time with SpO_2_ <90%. Other predefined endpoints included pain intensity, sedation level, subjective sleep quality, delirium within 5 days, non-delirium complications within 30 days, length of hospital stay, and all-cause 30-day mortality, as well as quality of life, cognitive function, and overall subjective sleep quality at 30 days after surgery.

For sleep structure and sleep-respiratory results, analyses were performed in patients who completed polysomnographic monitoring. For other perioperative outcomes, analyses were performed in the intent-to treat population, that is, all patients were analyzed in the group to which they were randomized.

For baseline and perioperative data, continuous data with a normal distribution were compared with independent sample *t*-tests; those with a non-normal distribution were compared with independent sample Mann–Whitney tests. Categorical data were compared with chi-square, continuity-corrected chi-square, or Fisher exact tests.

For the primary endpoint, the percentage of stage N2 sleep was compared with Mann–Whitney test; the difference between two medians and 95% CIs were calculated using the Hodges–Lehmann estimators. As exploratory analyses, the percentages of stage N2 sleep were also compared in the subgroups of patients stratified according to age (<65 years or ≥65 years), baseline PSQI (<6 points or ≥6 points), time of surgery (morning or afternoon/evening), and site of surgery (intrathoracic/upper abdominal or lower abdominal/spinal and extremital).

For other outcome analyses, continuous results with a non-normal distribution or ordinal data (sleep-structure parameters, subjective sleep quality, pain intensity, sedation level, quality of life, cognitive function, and overall subjective sleep quality) were compared with Mann–Whitney tests. The difference between two medians and 95% CIs were calculated using the Hodges–Lehmann estimators. Categorical variables (delirium within 5 days, non-delirium complications within 30 days, all-cause 30-day mortality) were analyzed with chi-square tests, chi-square tests with continuity correction, or Fisher exact tests; differences between groups were expressed as relative risk (RR) and 95% CIs. Time-to event variable (length of hospital stay) was analyzed with Kaplan–Meier survival analysis and log-rank test; univariable Cox proportional hazards model was used to calculate hazard ratio and 95% CI.

All tests were two-sided. *p*-values of <0.05 were considered statistically significant. Statistical analyses were performed with SPSS 26.0 software (IBM SPSS, Inc., Chicago, IL).

## 3 Results

### 3.1 Baseline and perioperative data

**Figure 1:**
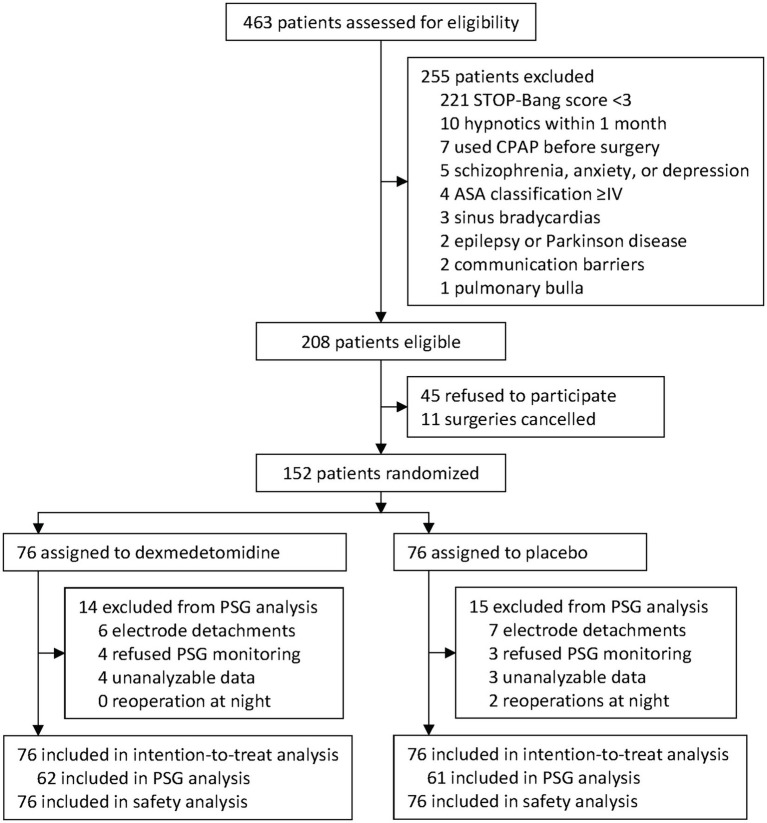
Trial diagram. STOP-Bang, the snoring, tiredness, observed apnea, high blood pressure-body mass index, age, neck circumference, and male gender. PSG, polysomnogram.

From 29 January 2021 to 20 September 2022, 463 patients were screened for inclusion. Of these, 208 were eligible, and 152 were enrolled and randomized to receive either dexmedetomidine (*n* = 76) or placebo (*n* = 76). All enrolled patients were included in the intention-to-treat and safety analyses. During the study period, 2 patients underwent reoperation on the night of the surgery, 7 patients refused sleep monitoring, and polysomnographic monitoring failed in 20 patients (13 had electrodes detached, and 7 gave unanalyzable data). Finally, 123 patients were included in the sleep architecture analysis (Figure 1). No emergency unblinding was needed during the study period.

Baseline characteristics were well-balanced between the two groups, except that dexmedetomidine group received more beta-blockers among all enrolled patients but fewer angiotensin-converting enzyme inhibitors/angiotensin receptor blockers among those included in sleep architecture analysis (Table 1; Supplementary Table S1). Perioperative variables were also well-balanced between the two groups, except that the dexmedetomidine group had lower mean airway pressure during mechanical ventilation among all enrolled patients. The median rate of dexmedetomidine administration (in the dexmedetomidine group) was 0.02 μg/kg/h in both all enrolled patients and those included in the sleep structure analysis (Table 2).

**Table 1:** Baseline data.

	All enrolled patients			Patients included in PSG analysis		
	Dexmedetomidine (*n* = 76)	Placebo (*n* = 76)	*p*-value	Dexmedetomidine (*n* = 62)	Placebo (*n* = 61)	*p*-value
Age, years	63 (58, 68)	62 (56, 66)	0.387	63 (58, 68)	61 (55, 67)	0.208
Male sex	54 (71%)	59 (78%)	0.353	42 (68%)	46 (75%)	0.346
Body mass index, kg m^−2^	25.7 ± 4.0	25.6 ± 3.2	0.928	25.9 ± 4.0	25.4 ± 3.3	0.441
Education, years	11 (8, 13)	11 (8, 13)	0.465	11 (8, 13)	11 (8, 13)	0.582
Preoperative history, diagnoses, and general status						
Preoperative comorbidity						
Hypertension	50 (66%)	52 (68%)	0.730	43 (69%)	45 (74%)	0.587
Coronary heart disease	5 (7%)	6 (8%)	0.754	4 (7%)	4 (7%)	>0.999
Previous stroke	6 (8%)	4 (5%)	0.513	6 (10%)	2 (3%)	0.273
Arrhythmia	4 (5%)	2 (3%)	0.681	4 (7%)	1 (2%)	0.365
COPD	4 (5%)	2 (3%)	0.681	4 (7%)	1 (2%)	0.365
Diabetes mellitus	19 (25%)	17 (22%)	0.703	16 (26%)	10 (16%)	0.201
Thyroid disease^a^	4 (5%)	2 (3%)	0.681	4 (7%)	1 (2%)	0.365
Liver dysfunction^b^	0 (0%)	1 (1%)	>0.999	0 (0%)	1 (2%)	0.496
Renal dysfunction^c^	0 (0%)	0 (0%)	—	0 (0%)	0 (0%)	—
Diagnosed OSA	0 (0%)	1 (1%)	>0.999	0 (0%)	1 (2%)	0.496
Chronic smoking^d^	26 (34%)	29 (38%)	0.613	24 (39%)	22 (36%)	0.762
Alcoholism^e^	14 (18%)	18 (24%)	0.426	13 (21%)	13 (21%)	0.963
Previous surgery	32 (42%)	27 (36%)	0.405	27 (44%)	22 (36%)	0.397
Preoperative medications						
Calcium channel blocker	28 (37%)	26 (34%)	0.735	26 (42%)	20 (33%)	0.294
Beta-blocker	16 (21%)	7 (9%)	**0.042**	14 (23%)	6 (10%)	0.055
ACEI/ARB	12 (16%)	22 (29%)	0.052	10 (16%)	20 (33%)	**0.031**
Aspirin	9 (12%)	8 (11%)	0.797	7 (11%)	7 (12%)	0.974
Subcutaneous insulin	3 (4%)	5 (7%)	0.719	3 (5%)	4 (7%)	0.717
Metformin	10 (13%)	10 (13%)	>0.999	8 (13%)	6 (10%)	0.592
Surgical diagnosis			0.779			0.802
Urogenital cancer^f^	44 (58%)	44 (58%)		37 (60%)	35 (57%)	
Gastrointestinal cancer^g^	11 (15%)	13 (17%)		8 (13%)	11 (18%)	
Other cancers^h^	3 (4%)	5 (7%)		3 (5%)	4 (7%)	
Non-cancer diseases^i^	18 (24%)	14 (18%)		14 (23%)	11 (18%)	
NYHA classification			0.561			0.570
I	74 (97%)	75 (99%)		60 (97%)	60 (98%)	
II	2 (3%)	1 (1%)		2 (3%)	1 (2%)	
ASA classification			0.893			0.846
I	1 (1%)	0 (0%)		1 (2%)	0 (0%)	
II	55 (72%)	56 (74%)		42 (68%)	44 (72%)	
III	20 (26%)	20 (26%)		19 (31%)	17 (28%)	
Preoperative laboratory tests						
Hematocrit, %	42.5 ± 3.7	42.7 ± 4.5	0.757	42.6 ± 3.8	42.2 ± 4.3	0.598
Albumin, g L^−1^	44.1 (40.7, 45.8)	43.6 (41.6, 45.7)	0.959	44.3 (41.6, 45.8)	43.5 (42.0, 45.7)	0.676
Alanine transaminase, IU L^−1^	17 (12, 26)	18 (13, 24)	0.483	17 (11, 25)	19 (13, 25)	0.274
Aspartate aminotransferase, IU L^−1^	20 (17, 24)	21 (18, 25)	0.335	20 (17, 24)	21 (18, 25)	0.176
Glucose, mmol L^−1^	5.7 (5.2, 6.3) [2]	5.7 (5.3, 6.6) [1]	0.461	5.7 (5.2, 6.4)	5.8 (5.3, 6.5) [1]	0.678
Sodium, mmol L^−1^	140.3 ± 1.9	140.5 ± 1.7	0.476	140.2 ± 1.9	140.6 ± 1.6	0.319
Potassium, mmol L^−1^	4.0 (3.7, 4.2)	3.9 (3.6, 4.1)	0.074	4.0 (3.7, 4.1)	3.9 (3.6, 4.1)	0.199
Creatinine, μmol L^−1^	80.7 (72.3, 89.2)	78.8 (69.7, 89.5) [1]	0.439	80.5 (70.1, 89.3)	78.1 (69.3, 88.0)	0.509
Bicarbonate, mmol L^−1^	29.2 (28.5, 30.1)	29.1 (28.5, 29.9)	0.531	29.3 (28.5, 30.0)	29.3 (28.4, 30.2)	0.740
Preoperative assessment, point						
Charlson comorbidity index^j^	4 (3, 5)	4 (3, 4)	0.583	4 (3, 5)	4 (3, 4)	0.117
Mini-Mental State Examination^k^	27 ± 2.2	27 ± 2.1	0.428	27 ± 2.3	28 ± 1.8	0.163
Pittsburgh Sleep Quality Index^l^	4 (2, 6)	3 (2, 5)	0.213	4 (2, 6)	3 (2, 5)	0.073
Pain intensity^m^	0 (0, 0)	0 (0, 0)	0.265	0 (0, 0)	0 (0, 0)	0.364
HADS-Depression^n^	0 (0, 0)	0 (0, 0)	0.961	0 (0, 0)	0 (0, 0)	0.783
HADS-Anxiety^n^	0 (0, 2)	0 (0, 2)	0.210	0 (0, 2)	0 (0, 2)	0.170
STOP-Bang score^o^	3 (3, 4)	3 (3, 4)	0.713	3 (3, 4)	3 (3, 4)	0.724
Preoperative delirium	0 (0%)	0 (0%)	>0.999	0 (0%)	0 (0%)	>0.999

Data are median (interquartile range), *n* (%), or mean ± SD. Numbers in square brackets indicate patients with missing data. *p*-values in bold indicate <0.05. PSG, polysomnography; COPD, chronic obstructive pulmonary disease; OSA, obstructive sleep apnea; ACEI, angiotensin-converting enzyme inhibitor; ARB, angiotensin receptor blocker; NYHA, New York Heart Association; ASA, American Society of Anesthesiologists; HADS, Hospital Anxiety and Depression Scale.

a Included hyperthyroidism, hypothyroidism, nodular goiter, Hashimoto’s thyroiditis, and thyroid adenoma.

b Serum alanine and/or aspartate transaminase higher than five times the upper normal limit.

c Creatinine concentration higher than 177 μmol/L.

d Smoking half a pack (10 cigarettes)/day for at least 1 year, either former or current smoker.

e Two drinks or more daily or weekly consumption of the equivalent of 150 mL of alcohol for at least 5 years.

f Include renal cancer, ureteral cancer, bladder cancer, and prostatic cancer. Also see Supplementary Table S1 for details.

g Included esophageal cancer, gastric cancer, colonic cancer, and rectal cancer. Also see Supplementary Table S1 for details.

h Include lung carcinoma and cholangiocarcinoma. Also see Supplementary Table S1 for details.

i Included adrenocortical adenoma, prolapse of lumbar intervertebral disc, and appendicular adenoma. Also see Supplementary Table S1 for details.

j According to the Charlson comorbidity index with age.

k Score ranges from 0 to 30, with higher score indicating better cognitive function.

l A self-rated questionnaire which assesses sleep quality and disturbances over a 1-month period; total scores range from 0 to 21, with higher score indicate worse sleep quality.

m An 11-point scale where 0 indicates no pain and 10 indicates the worst pain.

n Scores range from 0 to 21 for either depression or anxiety, with higher scores indicating more severe symptoms.

o The STOP-Bang questionnaire is a scoring model consisting of eight easily administered questions starting with the acronym STOP-Bang and is scored based on yes/no answers (score: 1/0). Total scores range from 0 to 8. A score of ≥3 has shown a high sensitivity for detecting obstructive sleep apnea.

**Table 2:** Intraoperative and postoperative data.

	All enrolled patients			Patients included in PSG analysis		
	Dexmedetomidine (*n* = 76)	Placebo (*n* = 76)	*p*-value	Dexmedetomidine (*n* = 62)	Placebo (*n* = 61)	*p*-value
Intraoperative data						
Duration of anesthesia, min	196 (140, 268)	206 (153, 271)	0.557	185 (133, 260)	194 (146, 269)	0.542
Type of anesthesia			0.276			0.139
General alone	18 (24%)	24 (32%)		13 (21%)	20 (33%)	
Combined regional-general	58 (76%)	52 (68%)		49 (79%)	41 (67%)	
Medications during anesthesia						
Use of midazolam	25 (33%)	27 (36%)	0.732	19 (31%)	20 (33%)	0.799
Dose of midazolam, mg	1.5 (1, 2)	1 (1, 2)	0.218	2 (1, 2)	1.5 (1, 2)	0.227
Use of propofol	76 (100%)	76 (100%)	>0.999	44 (100%)	41 (100%)	>0.999
Dose of propofol, mg	724 (500, 950)	715 (585, 897)	0.676	610 (461, 986)	708 (556, 886)	0.828
Use of etomidate	66 (87%)	63 (83%)	0.497	53 (86%)	51 (84%)	0.773
Dose of etomidate, mg	10 (10, 14)	12 (9, 14)	0.805	12 (10, 15)	10 (8, 14)	0.527
Use of remifentanil	66 (87%)	65 (86%)	0.814	54 (87%)	53 (87%)	0.972
Dose of remifentanil, μg	928 (629, 1,248)	966 (606, 1,238)	0.830	928 (585, 1,225)	905 (603, 1,190)	0.927
Use of sufentanil	76 (100%)	76 (100%)	>0.999	62 (100%)	61 (100%)	>0.999
Dose of sufentanil, μg	30 (25, 38)	30 (25, 45)	0.234	30 (20, 38)	30 (25, 45)	0.483
Use of rocuronium	74 (97%)	75 (99%)	>0.999	60 (97%)	60 (98%)	>0.999
Dose of rocuronium, mg	65 (50, 80)	60 (50, 80)	0.526	70 (50, 80)	60 (50, 80)	0.244
Use of cisatracurium	22 (29%)	24 (32%)	0.724	16 (26%)	16 (26%)	0.957
Dose of cisatracurium, mg	8 (3, 13)	9 (6, 14)	0.674	8 (3, 14)	9 (5, 12)	0.592
Use of sevoflurane inhalation	70 (92%)	69 (91%)	0.772	57 (92%)	56 (92%)	>0.999
Use of neostigmine	51 (67%)	56 (74%)	0.374	43 (69%)	43 (71%)	0.891
Use of esmolol	38 (50%)	29 (38%)	0.141	31 (50%)	22 (36%)	0.119
Use of vasopressors^a^	36 (47%)	32 (42%)	0.514	30 (48%)	26 (43%)	0.521
Use of vasodilators^b^	14 (18%)	8 (11%)	0.167	13 (21%)	6 (10%)	0.088
Use of NSAIDs^c^	62 (82%)	64 (84%)	0.667	48 (77%)	53 (87%)	0.171
Use of 5-HT3 receptor antagonists^d^	63 (83%)	67 (88%)	0.356	49 (79%)	55 (90%)	0.088
Use of methylprednisolone^e^	39 (51%)	32 (42%)	0.255	27 (44%)	24 (39%)	0.636
Use of other glucocorticoids^f^	29 (38%)	34 (45%)	0.410	28 (45%)	29 (48%)	0.791
Ventilation parameters						
Tidal volume, mL	499 ± 54	502 ± 53	0.778	498 ± 55	500 ± 53	0.850
Respiratory rate	13 (12, 14)	12 (12, 13)	0.112	13 (12, 14)	12 (12, 13)	0.063
Oxygen concentration, %	50 (50, 50)	50 (40, 50)	0.551	50 (45, 50)	50 (40, 50)	0.575
Mean airway pressure, cm H_2_O	17 (15, 20)	19 (15, 20)	**0.037**	17 (15, 20)	19 (15, 21)	0.066
Peak airway pressure, cm H_2_O	20 (17, 22)	20 (17, 24)	0.115	20 (18, 22)	20 (17, 24)	0.342
PEEP, cm H_2_O	4 (0, 4)	4 (0, 5)	0.994	4 (0, 4)	4 (0, 5)	0.627
Arterial blood gas						
Minimum hemoglobin, g/dL	13.0 ± 1.6 [8]	12.6 ± 1.9 [10]	0.201	13.0 ± 1.6 [3]	12.5 ± 1.8 [9]	0.116
Maximum blood glucose, mmol/L	5.8 (5.3, 6.7) [6]	5.9 (5.1, 7.4) [5]	0.620	5.7 (5.3, 6.6) [2]	5.9 (5.1, 7.3) [5]	0.603
Maximum lactate, mmol/L	1.1 (0.8, 1.4) [7]	1.0 (0.8, 1.4) [5]	0.296	1.1 (0.8, 1.4) [3]	1.0 (0.8, 1.4) [5]	0.462
Total volume infused, mL	1,600 (1,500, 2,350)	1,600 (1,500, 2,338)	0.712	1,600 (1,500, 2,150)	1,600 (1,500, 2,100)	0.657
Crystalloids, mL	1,500 (1,100, 1,838)	1,600 (1,100, 1,800)	0.438	1,500 (1,100, 1,600)	1,500 (1,100, 1,800)	0.400
Use of artificial colloids	40 (53%)	39 (51%)	0.871	32 (52%)	31 (51%)	0.930
Estimated blood loss, mL	50 (20, 100)	50 (20, 100)	0.843	50 (20, 80)	50 (20, 100)	0.786
Blood transfusion	3 (4%)	4 (5%)	>0.999	3 (5%)	4 (7%)	0.717
Urine output, mL	300 (200, 500) [16]	300 (150, 600) [13]	0.988	300 (200, 500) [14]	275 (150, 638) [9]	0.698
Site of surgery			0.953			0.965
Intrathoracic	3 (4%)	4 (5%)		3 (5%)	3 (5%)	
Upper abdominal	39 (49%)	41 (54%)		31 (48%)	33 (54%)	
Lower abdominal	25 (36%)	23 (30%)		22 (37%)	19 (31%)	
Spinal and extremital	9 (12%)	8 (11%)		6 (10%)	6 (10%)	
Type of surgery			0.403			0.215
Open	12 (16%)	16 (21%)		8 (13%)	13 (21%)	
Mini-invasive^g^	64 (84%)	60 (79%)		54 (87%)	48 (79%)	
Duration of surgery, min	131 (80, 184)	146 (93, 191)	0.317	112 (63, 183)	136 (92, 191)	0.315
Postoperative data						
Admission to the ICU	6 (8%)	4 (5%)	0.513	5 (8%)	1 (2%)	0.207
Duration of PSG monitoring, min	—	—	—	540 (540, 540)	540 (540, 540)	0.150
Consumed volume of PCIA, mL	76 (60, 92)	78 (57, 99)	0.574	76 (59, 91)	76 (52, 97)	0.887
Duration of PCIA, h	72 (60, 84)	72 (52, 90)	0.534	72 (58, 84)	72 (48, 89)	0.998
Rate of dexmedetomidine, μg kg^−1^ h^−1^	0.02 (0.02, 0.02)	—	—	0.02 (0.02, 0.02)	—	—
Use of high-flow nasal cannula	66 (87%)	65 (86%)	0.814	57 (92%)	55 (90%)	0.731
Supplemental analgesics within 5 days	50 (66%)	43 (57%)	0.244	39 (63%)	35 (57%)	0.531
Use of NSAIDs^h^	47 (62%)	39 (51%)	0.190	37 (60%)	32 (53%)	0.420
Use of acetaminophen	12 (16%)	13 (17%)	0.827	8 (13%)	10 (16%)	0.584
Use of opioids^i^	12 (16%)	13 (17%)	0.827	8 (13%)	10 (16%)	0.584
Use of other sedatives within 5 days^j^	4 (5%)	5 (7%)	>0.999	4 (7%)	1 (2%)	0.365

Data are median (interquartile range), *n* (%), or mean ± SD. Numbers in square brackets indicate patients with missing data. *p*-value in bold indicates <0.05. PSG, polysomnography; NSAIDs, non-steroid anti-inflammatory drugs; 5-HT3, 5-hydroxytryptamine type 3; PEEP, positive end-expiratory pressure; ICU, intensive care unit; PCIA, patient-controlled intravenous analgesia.

a Included ephedrine, phenylephrine, metaraminol, and dopamine.

b Included nicardipine and urapidil.

c Included flurbiprofen axetil (50–100 mg) and parecoxib (40 mg).

d Included tropisetron (5 mg) and ondansetron (4 mg).

e Intravenous injection, 40 mg each time.

f Mainly dexamethasone (5–10 mg) or hydrocortisone (50 mg), for intravenous injection.

g Included laparoscopic and thoracoscopic surgeries.

h Included intravenous flurbiprofen axetil (50–200 mg d^−1^) or parecoxib (80 mg d^−1^), or oral loxoprofen (180 mg d^−1^).

i Included subcutaneous morphine 10 mg and oral oxycodone 5 mg.

j Included oral diazepam (2.5 mg) or estazolam (1 mg) for sleep promotion. These drugs were used after the night of surgery, so polysomnography monitoring was not affected.

### 3.2 Sleep structure analysis

The percentage of stage N2 sleep was higher in patients given dexmedetomidine (median 69%; interquartile range, 46 to 79%) than in those given placebo (median 51%; interquartile range, 27 to 76%; median difference, 10%; 95% CI, 1 to 21%; *p* = 0.029). Stratified analyses showed that the percentage of N2 sleep was higher with dexmedetomidine than with placebo in the subgroup of patients aged under 65 years (median difference, 13%; 95% CI, 2 to 29%; *p* = 0.028), who had better baseline sleep quality (PSQI <6; median difference, 13%; 95% CI, 3 to 25%; *p* = 0.018), and who underwent surgery in the morning (median difference, 16%; 95% CI, 2 to 31%; *p* = 0.034; Table 3).

**Table 3:** Sleep architecture analysis.

	Dexmedetomidine (*n* = 62)	Placebo (*n* = 61)	Median difference or RR (95% CI)^a^	*p*-value
Primary endpoint				
Percentage of N2 sleep, %	69 (46, 79)	51 (27, 76)	10 (1 to 21)	**0.029**
Stratified analysis of N2 sleep, %				
According to patients’ age				
<65 years	69 (46, 79) (*n* = 36)	47 (10, 74) (*n* = 42)	13 (2 to 29)	**0.028**
≥65 years	68 (45, 79) (*n* = 26)	57 (44, 79) (*n* = 19)	4 (−10 to 17)	0.543
According to baseline PSQI				
<6 points	68 (46, 80) (*n* = 45)	49 (19, 74) (*n* = 50)	13 (3 to 25)	**0.018**
≥6 points	73 (46, 79) (*n* = 17)	71 (28, 83) (*n* = 11)	−3 (−20 to 23)	0.890
According to time of surgery				
Morning surgery (8 am–2 pm)	70 (52, 84) (*n* = 25)	51 (22, 72) (*n* = 21)	16 (2 to 31)	**0.034**
Afternoon/evening surgery (≥2 pm)	64 (41, 78) (*n* = 37)	51 (27, 78) (*n* = 40)	6 (−5 to 21)	0.301
According to site of surgery				
Intrathoracic/upper abdominal	72 (46, 82) (*n* = 34)	50 (29, 79) (*n* = 36)	9 (−3 to 24)	0.152
Lower abdominal/spinal and extremital	60 (46, 77) (*n* = 28)	52 (19, 72) (*n* = 25)	13 (−3 to 30)	0.094
Secondary endpoints				
Total sleep time, min^b^	175 (111, 249)	187 (86, 278)	−1 (−43 to 41)	0.964
Sleep efficiency, %^c^	34 (21, 47)	36 (17, 53)	−1 (−9 to 7)	0.828
Duration of N1 sleep, min	51 (23, 84)	47 (24, 98)	−5 (−21 to 10)	0.511
Percentage of N1 sleep, %	29 (16, 49)	44 (21, 69)	−10 (−20 to −1)	**0.042**
Duration of N2 sleep, min	112 (47, 175)	89 (27, 163)	16 (−13 to 45)	0.293
Duration of N3 sleep, min	0 (0, 0)	0 (0, 0)	0 (0 to 0)	0.142
Percentage of N3 sleep, %	0 (0, 0)	0 (0, 0)	0 (0 to 0)	0.139
Presence of N3 sleep	9 (15%)	4 (7%)	RR = 2.21 (0.72 to 6.81)	0.151
Duration of REM sleep, min	0 (0, 3)	0 (0, 1)	0 (0 to 0)	0.328
Percentage of REM sleep, %	0 (0, 2)	0 (0, 1)	0 (0 to 0)	0.388
Presence of REM sleep	23 (37%)	16 (26%)	RR = 1.41 (0.83 to 2.41)	0.195
Sleep fragmentation index, times per hour^d^	7.0 (4.4, 10.9)	6.1 (3.6, 10.5) [1]	0.3 (−1.2 to 1.9)	0.684
Exploratory analysis				
AHI, events per hour^e^	4 (1.1, 8.8) [4]	2.5 (0.8, 7.1) [5]	0.8 (−0.5 to 2.5)	0.259
REM-AHI, events per hour^f^	0 (0.0, 0.0) [4]	0.0 (0.0, 0.0) [5]	0.0 (0.0 to 0.0)	0.190
NREM-AHI, events per hour^g^	0.7 (0.0, 3.5) [4]	0.3 (0.0, 2.2) [5]	0.0 (0.0 to 0.5)	0.308
Apnea index, events per hour^h^	0.7 (0.0, 3.8) [4]	0.4 (0.0, 2.2) [5]	0.0 (0.0 to 0.6)	0.300
Obstructive apnea index, events per hour^i^	0.7 (0.0, 3.8) [4]	0.4 (0.0, 1.9) [5]	0.0 (0.0 to 0.6)	0.356
Central apnea index, events per hour^j^	0.0 (0.0, 0.0) [4]	0.0 (0.0, 0.0) [5]	0.0 (0.0 to 0.0)	0.323
Mixed apnea index, events per hour^k^	0.0 (0.0, 0.0) [4]	0.0 (0.0, 0.0) [5]	0.0 (0.0 to 0.0)	0.429
Hypopnea index, events per hour^l^	1.9 (0.2, 3.6) [4]	0.8 (0.2, 3.1) [5]	0.4 (−0.1 to 1.3)	0.221
Respiratory arousal index, events per hour^m^	0.9 (0, 2.9) [5]	0.3 (0.0, 1.6) [6]	0.0 (0.0 to 0.7)	0.209
Oxygen desaturation index, events per hour^n^	1.1 (0.0, 5.5) [3]	0.9 (0.0, 3.4) [5]	0.0 (−0.1 to 0.8)	0.380
Percentage of time with SpO_2_ <90%, %^o^	0 (0, 0) [3]	0 (0, 0) [5]	0 (0 to 0)	0.813
Lowest SpO_2_, %^p^	94 (90, 96) [3]	94 (90, 97) [5]	0 (−2 to 1)	0.747
Mean nocturnal SpO_2_, %	99 (97, 100) [3]	99 (98, 100) [5]	0 (−1 to 0)	**0.024**

Data are median (interquartile range) or *n* (%). Numbers in square brackets indicate patients with missing data. *p*-values in bold indicate <0.05. RR, relative risk; N2/1/3, nonrapid eye movement stage 2/1/3; PSQI, Pittsburgh Sleep Quality Index; REM, rapid eye movement; NREM, nonrapid eye movement; RR, relative risk; AHI, apnea-hypopnea index.

a Calculated as dexmedetomidine group vs. or minus placebo group.

b Total time spent in any sleep stage during the monitoring period, i.e., from 9 pm on the night of surgery to 6 am the next morning.

c The ratio between the total sleep time and the total recording time and expressed as percentage.

d The average number of arousals and awakenings per hour of sleep.

e Average number of apnea and hypopnea episodes per hour.

f Apnea hypopnea index during REM sleep.

g Apnea hypopnea index during NREM sleep.

h Average number of apnea episodes per hour.

i Average number of apnea episodes per hour that occurred secondary to airway collapse with subsequent blockage of the upper airway during sleep.

j Average number of apnea episodes per hour that occurred secondary to lack of signal from the brain to breath.

k Apnea episodes with characteristics of both obstructive and central apnea.

l Average number of hypopnea episodes per hour.

m Average hourly sleep arousals due to respiratory events.

n Average number per hour of episodes with 3% or greater desaturation and lasting 10 s or longer.

o The percentage of cumulative time spent with SpO_2_ <90% during sleep.

p The lowest SpO_2_ value during sleep.

Among secondary endpoints, the percentage of stage N1 sleep was lower with dexmedetomidine than with placebo (median difference, −10%; 95% CI, −20% to −1%; *p* = 0.042); other sleep structure parameters did not differ significantly between the two groups. In exploratory analyses, patients given dexmedetomidine had a lower mean nocturnal SpO_2_, but the difference was not clinically important (median difference, 0%; 95% CI, −1 to 0%; *p* = 0.024); other sleep-respiratory parameters did not differ between groups (Table 3).

### 3.3 Other postoperative outcomes

**Figure 2:**
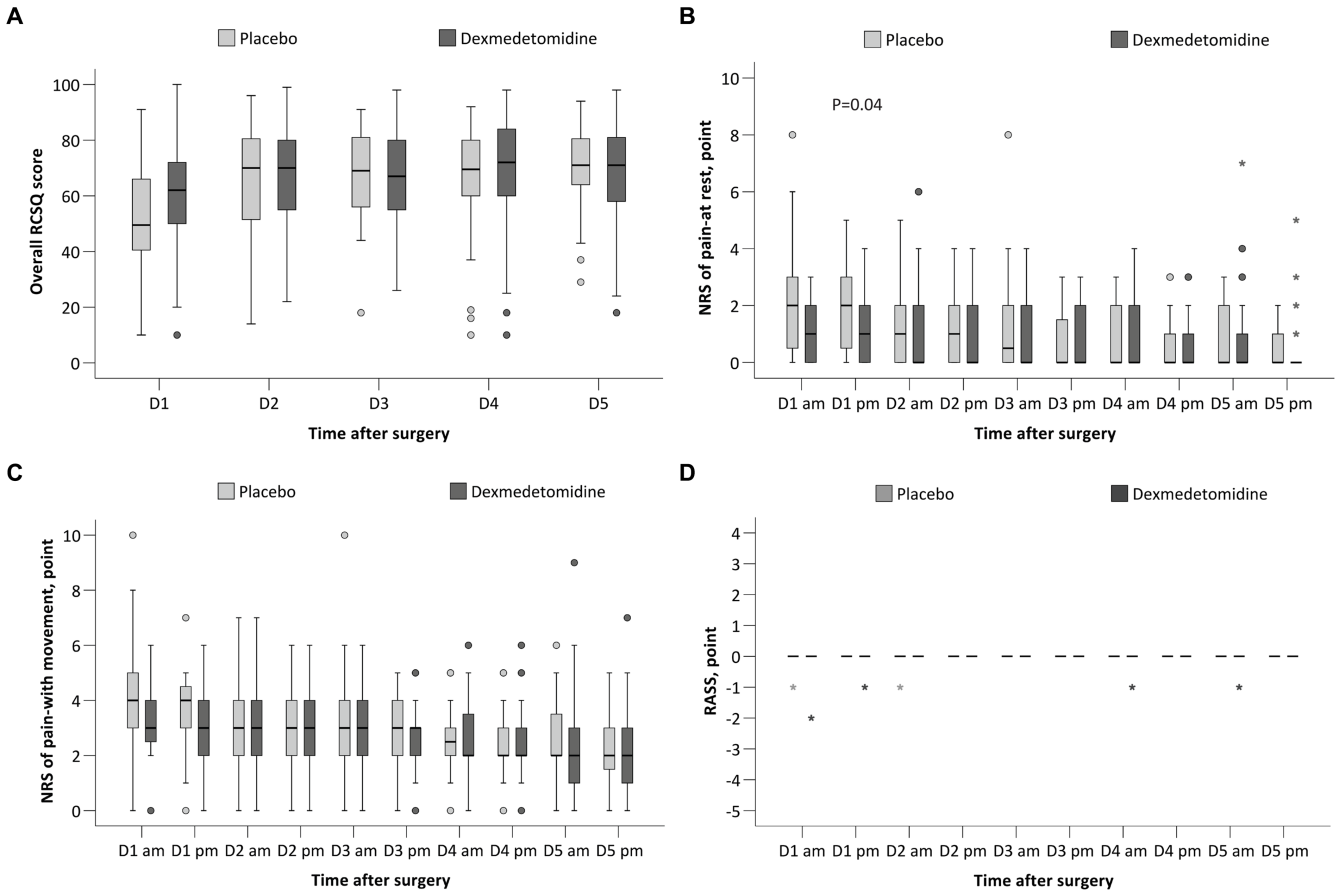
Comparison of the RCSQ score **(A)**, NRS of pain at rest **(B)**, NRS of pain with movement **(C)**, and the RASS **(D)** between the two groups. RCSQ, the Richards-Campbell Sleep Questionnaire, a self-reported measure of subjective sleep quality in 5 items; the score of each item ranges from 0 to 100, with a higher score indicating better sleep. The mean score of the five items represents the overall sleep quality. NRS, numeric rating scale, an 11-point scale where 0 indicates no pain or the best sleep and 10 indicates the worst pain or the worst sleep. RASS, the Richmond Agitation-Sedation Scale; the score ranges from −5 (unarousable) to +4 (combative), and 0 indicates alert and calm. The box and whiskers plots show medians, interquartile ranges, and outer ranges; individual points indicate mild outliers (o, outside 1.5 times of interquartile range) and extreme outliers (*, outside 3 times of interquartile range).

The overall RCSQ score on the night of surgery was slightly higher (better) in patients given dexmedetomidine, but the difference was not statistically significant (median difference, 6; 95% CI, 0 to 13; *p* = 0.060; Figure 2A); the overall RCSQ score at other timepoints did not differ between groups (Table 4). Regarding the individual RCSQ items, the scores of sleep latency (median difference, 10; 95% CI, 0 to 15; *p* < 0.050) and awakenings (median difference, 10; 95% CI, 0 to 10; *p* = 0.028) were higher (better) with dexmedetomidine on the night of surgery and were clinically meaningful (Supplementary Table S2).

**Table 4:** Postoperative outcomes.

	Dexmedetomidine (*n* = 76)	Placebo (*n* = 76)	Estimated differences (95% CI)^a^	*p*-value
Overall RCSQ score, point^a^				
Day 1	60 (49, 72)	54 (38, 70)	Median *D* = 6 (0 to 13)	0.060
Day 2	71 (56, 81)	73 (52, 81)	Median *D* = −2 (−8 to 4)	0.612
Day 3	69 (55, 81) [3]	72 (56, 82) [1]	Median *D* = −2 (−7 to 4)	0.537
Day 4	72 (59, 82) [7]	74 (62, 82) [6]	Median *D* = −1 (−7 to 4)	0.629
Day 5	71 (58, 82) [23]	71 (64, 81) [20]	Median *D* = 0 (−6 to 5)	0.851
Delirium within 5 days	1 (1%)	1 (1%)	RR = 1.00 (0.06 to 15.7)	>0.999
Non-delirium complications within 30 days^b^	22 (26%)	21 (28%) [1]	RR = 0.94 (0.56 to 1.59)	0.816
Arrhythmia^c^	1 (1%)	1 (1%)	RR = 1.00 (0.06 to 15.7)	>0.999
Acute kidney injury^d^	13 (17%) [1]	15 (20%) [1]	RR = 0.87 (0.44 to 1.70)	0.675
Intestinal obstruction^e^	2 (3%)	1 (1%)	RR = 2.00 (0.19 to 21.6)	>0.999
Reoperation^f^	0 (0%)	2 (3%)	—	0.497
Death within 30 days	0 (0%)	0 (0%)	—	>0.999
Length of hospital stay after surgery, day	6 (4, 7)	6 (4, 8)	HR = 0.90 (0.65 to 1.24)	0.516
PSQI at 30 days, point^g^	4 (2, 6)	3 (2, 5) [1]	Median *D* = 1 (0 to 1)	0.135
Quality of life at 30 days, point^h^				
Physiology domain	68 (64, 78)	71 (64, 79) [1]	Median *D* = −0 (−4 to 4)	0.612
Psychological domain	58 (55, 63)	58 (58, 63) [1]	Median *D* = 0 (−4 to 0)	0.100
Social domain	67 (50, 75)	67 (50, 75) [1]	Median *D* = 0 (0 to 0)	0.985
Environment domain	63 (59, 72)	63 (59, 72) [1]	Median *D* = 0 (0 to 3)	0.399
Cognitive function at 30 days, point^i^	33 ± 4	33 ± 3 [1]	Mean *D* = 0.1 (−1 to 1)	0.809

Data are median (interquartile range), *n* (%), or mean ± SD. Numbers in square brackets indicate patients with missing data. RCSQ, Richards-Campbell Sleep Questionnaire; D, difference; RR, relative risk; HR, hazard ratio; PSQI, Pittsburgh Sleep Quality Index.

a Subjective sleep quality in the last night after surgery was assessed with the RCSQ. The RCSQ is a five-item questionnaire. Responses are recorded on a 100 mm visual analog scale, with higher scores representing better sleep. The mean of these five items represents the overall RCSQ score.

b Generally defined as new-onset medical conditions that were deemed harmful to patients’ recovery and required medical intervention, i.e., grade II or higher on the Clavien-Dindo classification.

c Diagnosed by 12-lead ECG and required antiarrhythmic drugs.

d Diagnosed according to the KIDGO creatinine criteria, that is the presence and any of the following condition: (1) serum creatinine increase ≥0.3 mg/dL (≥26.5 μmoL/L) within 48 h; or (2) increase of serum creatinine ≥1.5 times of basal value within 7 days of onset.

e Disappearance of bowel sounds, inability to exhaust, abdominal pain or abdominal distension, which suggested the disappearance of intestinal movement until the fifth day after surgery.

f Any unplanned surgical re-intervention following surgery.

g Score ranges from 0 to 21, with a higher score indicating worse sleep quality.

h Assessed with the World Health Organization Quality of Life-brief version. The score of each domain ranges from 0 to 100, with higher score indicating better function.

i Assessed with the Telephone Interview of Cognition Scale-modified. The score ranges from 0 to 50, with higher score indicating better cognitive function.

The NRS pain score at rest was lower in the dexmedetomidine group in the afternoon of postoperative day 1 (median difference, 0; 95% CI, −1 to 0; *p* = 0.038), but the difference was not clinically meaningful (Figure 2B and Supplementary Table S3). The NRS pain score with movement at 10 timepoints across the first 5 postoperative days did not differ between the two groups (Figure 2C and Supplementary Table S3). The RASS scores at 10 timepoints were all 0 and did not differ between groups (Figure 2D and Supplementary Table S4).

Other postoperative outcomes, including delirium within 5 days, non-delirium complications within 30 days, length of hospital stay after surgery, as well as Pittsburgh Sleep Quality Index, quality of life, and cognitive function at 30 days, did not differ between the two groups. No patient died within 30 days (Table 4).

### 3.4 Safety outcomes

Adverse events within 48 h did not differ between the two groups. No patient developed bradycardia, respiratory depression, or excessive sedation. No severe adverse events occurred during the study period (Table 5).

**Table 5:** Adverse events within 48 h.

	Dexmedetomidine (*n* = 76)	Placebo (*n* = 76)	*p*-value
Study drug infusion <24 h^a^	3 (4%)	4 (5%)	>0.999
Hypotension^b^	0 (0%)	1 (1%)	>0.999
Intervention for hypotension	0 (0%)	0 (0%)	>0.999
Hypertension^c^	1 (1%)	2 (3%)	>0.999
Intervention for hypertension	1 (1%)	2 (3%)	>0.999
Bradycardia^d^	0 (0%)	0 (0%)	>0.999
Intervention for bradycardia	0 (0%)	0 (0%)	>0.999
Tachycardia^e^	0 (0%)	2 (3%)	0.497
Intervention for tachycardia	0 (0%)	1 (1%)	>0.999
Respiratory depression^f^	0 (0%)	0 (0%)	>0.999
Intervention for respiratory depression	0 (0%)	0 (0%)	>0.999
Excessive sedation^g^	0 (0%)	0 (0%)	>0.999
Postoperative nausea and vomiting	16 (21%)	19 (25%)	0.563
Intervention for nausea/vomiting	6 (8%)	8 (11%)	0.575

Data are *n* (%).

a Patient-controlled analgesia was interrupted due to postoperative nausea and vomiting. These patients were included in sleep architecture analysis because study drug infusion continued during the period of polysomnographic monitoring.

b Systolic blood pressure <90 mmHg or >30% lower than baseline.

c Systolic blood pressure >180 mmHg or >30% higher than baseline.

d Heart rate <40 beats per minute.

e Heart rate >100 beats per minute.

f Respiratory frequency <10 breath per minute.

g Richmond Agitation-Sedation Scale ≤−3.

## 4 Discussion

Our results showed that, for postoperative patients at high risk of OSA, mini-dose dexmedetomidine supplemented analgesia improved sleep architecture as manifested by increased percentage of stage N2 sleep and decreased percentage of stage N1 sleep; it also slightly improved subjective sleep quality although not statistically significant, without increasing adverse events.

The harmful impact of undiagnosed/untreated OSA has been increasingly recognized (Sun et al., 2022; Chan et al., 2019). The Society of Anesthesia and Sleep Medicine recommends routine preoperative screening for OSA in adult patients (Chung et al., 2016). When compared with patients at low risk of OSA, those at high risk of OSA as assessed with the STOP-Bang had a higher risk of postoperative complications and a longer length of hospital stay (Nagappa et al., 2017). In the present trial, we enrolled patients with a STOP-Bang score ≥3 and a serum bicarbonate level ≥28 mmol/L; the specificity of this combined threshold in predicting patients at high risk of moderate-to-severe OSA has been validated previously (Chung et al., 2013).

In the present study, we provided HFNC for all enrolled patients because of the following considerations. First, oxygen therapy should be provided for postoperative OSA patients because it may improve oxygenation and decrease AHI (Liao et al., 2017). Second, none of our patients received CPAP before surgery, whereas HFNC is considered a more acceptable substitute for CPAP (Yan et al., 2022). Indeed, 86% of our patients accepted HFNC during the study period, much higher than the reported acceptance rate of CPAP in postoperative patients (45 to 60%) (Suen et al., 2021). Previous studies showed that use of HFNC reduces AHI in OSA patients (Yan et al., 2022). Third, as a sedative, dexmedetomidine has the potential to aggravate the respiratory status of OSA patients. Whereas, in ICU patients receiving HFNC, sedative dose dexmedetomidine improved sleep quantity without increasing adverse events (Ueno et al., 2022).

During the study period, dexmedetomidine was provided as a supplement to PCIA. As a result, dexmedetomidine was administered at a median rate of 0.02 μg/kg/h. We adopted this dosing regimen because our previous studies showed that this mini-dose dexmedetomidine improved sleep quality without producing any sedation (Hong et al., 2021; Zhang et al., 2022), and we attempted to avoid deepening sedation in our patients. In accordance with the previous results (Zhang et al., 2022), we also found that the percentage of stage N2 sleep was increased by 10%, and accordingly, the percentage of stage N1 sleep was decreased by 10% during the night of surgery. However, N3 sleep did not differ significantly between the two groups. This weakened the effects of intervention on other outcomes because N3 sleep is important for postoperative recovery, but is understandable since the effect of dexmedetomidine is dose-dependent (Akeju et al., 2018) and we adopted a mini-dose regimen. Stratified analyses showed that the effect mini-dose dexmedetomidine was more prominent in the subgroup of patients aged younger than 65 years, had better preoperative sleep quality, or underwent surgery in the morning. These phenomenon may also be attributed to the facts that sleep structure deterioration was more severe in older patients (Butris et al., 2023), poor sleepers, and in those following surgery in the afternoon or evening (Song et al., 2020), and the sleep-promoting effect of mini-dose dexmedetomidine is weak (Akeju et al., 2018).

Different from previous studies (Chen et al., 2017; Wu et al., 2016; Zhang et al., 2022), the mini-dose dexmedetomidine did not significantly improve other sleep structure parameters and subjective sleep quality in our patients. Reasons leading to this discrepancy may include the following. First, the dose of dexmedetomidine is too small (Akeju et al., 2018). Most previous studies administered a dexmedetomidine dose higher than ours (Chen et al., 2017; Wu et al., 2016), but at a risk of producing sedation (Chen et al., 2017; Feng et al., 2019). In our recent study, the same mini-dose dexmedetomidine improved sleep structure but not subjective sleep quality (Zhang et al., 2022). Second, all our patients were provided with HFNC which, although improved oxygenation, might also have interfered with sleep (Nakanishi et al., 2020). Despite these, we note that the scores of sleep latency and awakenings of the individual RCSQ items were better with dexmedetomidine on the first night after surgery, indicating that subjective sleep quality was improved to some degree. In the present study, we did not find clinically important differences in sleep-respiratory parameters between the two groups. This may also be attributed to the above reasons. Furthermore, we did not monitor the most severe breathing disturbances during sleep which is reported to occur on the third night after surgery (Chung et al., 2014).

Among our patients, mini-dose dexmedetomidine as a supplement to opioid PCIA did not improve early postoperative analgesia to a clinically significant degree. This was different from many previous studies (Chen et al., 2017; Feng et al., 2019; Hong et al., 2021), but was consistent with our recent findings using a similar mini-dose regimen (Zhang et al., 2022). We again did not find that mini-dose dexmedetomidine produced significant sedation (Hong et al., 2021; Zhang et al., 2022). These results are also predictable because both analgesic and sedative effects of dexmedetomidine are dose-dependent (Mo and Zimmermann, 2013; Wu et al., 2016; Su et al., 2016; Hong et al., 2021; Zhang et al., 2022). Furthermore, our dosing regimen did not change other perioperative outcomes including PSQI at 30 days, nor did it increase adverse events.

There are some limitations worth mentioning. First, 19% (29/152) of our patients were excluded from sleep structure analysis. The exclusion was mainly due to technical reasons (electrode detachment, interference, or reoperation) and was comparable between the two groups, but may produce bias in our sleep structure results. Second, we only monitored polysomnograms during the first night after surgery. However, the severity of postoperative sleep disturbances is the greatest at this night, and it is therefore the most important period to monitor sleep and provide intervention (Chung et al., 2014). Third, 7% (10/152) of our patients were transferred to the ICU, and 6 of them (4% of all patients) completed PSG monitoring. The ICU environment might have interfered with sleep quality. However, excluding these patients did not change our results; the percentage of N2 sleep remained higher with dexmedetomidine than with placebo (median difference, 11%; 95% CI, 2 to 22%; *p* = 0.015). Lastly, our sample size is insufficient to detect differences in secondary outcomes, including subjective sleep quality and pain intensity.

In summary, for patients at high risk of OSA after surgery, supplementing intravenous analgesia with mini-dose dexmedetomidine marginally improved sleep quality without producing adverse effects. Future studies are required to find optimal intervention for and clinical significance of sleep promotion in this patient population.

## Data Availability

The original contributions presented in the study are included in the article/Supplementary material, further inquiries can be directed to the corresponding author.

## Author’s note

Presentation: Preliminary data for this study were presented as a poster during the 18th World Congress of Anaesthesiologists (WCA2024), 3 to 7 March 2024, Singapore.
