# Supplementary material for: Impact of mini-dose dexmedetomidine supplemented analgesia on sleep structure in patients at high risk of obstructive sleep apnea: a pilot trial
Source: Front Neurosci. 2024 Oct 2;18:1426729. doi: 10.3389/fnins.2024.1426729 (PMC11480026; doi:10.3389/fnins.2024.1426729)
Supplement: Supplementary file 1 [file Data_Sheet_1.docx]

**Supplemental Table S1.** Baseline surgical diagnosis.

|  | **All enrolled patients** | | | **Patients included in PSG analysis** | | |
| --- | --- | --- | --- | --- | --- | --- |
|  | **Dexmedetomidine (n=76)** | **Placebo (n=76)** | ***P* value** | **Dexmedetomidine (n=62)** | **Placebo (n=61)** | ***P* value** |
| Urogenital cancer |  |  | 0.716 |  |  | 0.442 |
| Renal cancer | 25 (57%) | 29 (66%) |  | 20 (54%) | 24 (69%) |  |
| Ureteral cancer | 3 (7%) | 1 (2%) |  | 2 (5%) | 1 (3%) |  |
| Bladder cancer | 4 (9%) | 4 (9%) |  | 3 (8%) | 4 (11%) |  |
| Prostatic cancer | 12 (27%) | 10 (23%) |  | 12 (32%) | 6 (17%) |  |
| Gastrointestinal cancer |  |  | >0.999 |  |  | 0.715 |
| Esophageal cancer | 0 (0%) | 0 (0%) |  | 0 (0%) | 0 (0%) |  |
| Gastric cancer | 3 (27%) | 3 (23%) |  | 2 (25%) | 2 (18%) |  |
| Colonic cancer | 4 (36%) | 6 (46%) |  | 2 (25%) | 5 (45%) |  |
| Rectal cancer | 4 (36%) | 4 (31%) |  | 4 (50%) | 4 (36%) |  |
| Other cancers |  |  | >0.999 |  |  | >0.999 |
| Lung cancer | 3 (100%) | 4 (80%) |  | 3 (100%) | 3 (75%) |  |
| Cholangiocarcinoma | 0 (0%) | 1 (20%) |  | 0 (0%) | 1 (25%) |  |
| Non-cancer diseases |  |  | >0.999 |  |  | >0.999 |
| Adrenocortical adenoma | 8 (44%) | 6 (43%) |  | 7 (50%) | 5 (45%) |  |
| Prolapse of lumbar intervertebral disc | 9 (50%) | 8 (57%) |  | 6 (43%) | 6 (55%) |  |
| Appendicular adenoma | 1 (6%) | 0 (0%) |  | 1 (7%) | 0 (0%) |  |

Data are n (%). PSG, polysomnography.

**Supplement Table S2.** Postoperative individual results of Richards-Campbell Sleep Questionnaire.^a^

|  | **Dexmedetomidine (n=76)** | **Placebo (n=76)** | **Median difference (95% CI) ^b^** | ***P* value** |
| --- | --- | --- | --- | --- |
| Sleep depth (point) |  |  |  |  |
| Day 1 | 60 (40, 70) | 50 (40, 70) | 10 (0 to 10) | 0.067 |
| Day 2 | 70 (51, 80) | 70 (50, 85) | 0 (-10 to 5) | 0.566 |
| Day 3 | 70 (55, 80) [3] | 75 (60, 80) [1] | 0 (-10 to 5) | 0.435 |
| Day 4 | 70 (60, 80) [7] | 73 (60, 86) [6] | 0 (-10 to 5) | 0.779 |
| Day 5 | 70 (55, 80) [23] | 70 (61, 80) [20] | 0 (-10 to 0) | 0.447 |
| Sleep latency (point) |  |  |  |  |
| Day 1 | 70 (50, 80) | 60 (40, 80) | 10 (0 to 15) | **<0.050** |
| Day 2 | 75 (60, 84) | 80 (60, 90) | 0 (-10 to 0) | 0.347 |
| Day 3 | 70 (53, 85) [3] | 80 (60, 80) [1] | 0 (-10 to 5) | 0.497 |
| Day 4 | 70 (58, 88) [7] | 75 (60, 85) [6] | 0 (-10 to 5) | 0.481 |
| Day 5 | 70 (60, 83) [23] | 70 (66, 80) [20] | 0 (-10 to 5) | 0.670 |
| Awakenings (point) |  |  |  |  |
| Day 1 | 50 (40, 70) | 50 (30, 60) | 10 (0 to 10) | **0.028** |
| Day 2 | 60 (50, 80) | 68 (50, 80) | 0 (-10 to 5) | 0.796 |
| Day 3 | 65 (50, 80) [3] | 70 (50, 80) [1] | 0 (-10 to 5) | 0.632 |
| Day 4 | 70 (50, 80) [7] | 68 (50, 80) [6] | 0 (-10 to 5) | 0.847 |
| Day 5 | 70 (60, 80) [23] | 70 (60, 75) [20] | 0 (-5 to 10) | 0.749 |
| Returning to sleep (point) |  |  |  |  |
| Day 1 | 70 (50, 80) | 60 (40, 80) | 5 (0 to 10) | 0.098 |
| Day 2 | 70 (60, 80) | 80 (55, 85) | 0 (-10 to 0) | 0.392 |
| Day 3 | 70 (60, 85) [3] | 80 (60, 90) [1] | 0 (-10 to 0) | 0.303 |
| Day 4 | 75 (60, 85) [7] | 80 (65, 90) [6] | 0 (-10 to 0) | 0.288 |
| Day 5 | 70 (60, 85) [23] | 70 (60, 84) [20] | 0 (-5 to 5) | 0.983 |
| Overall sleep quality (point) |  |  |  |  |
| Day 1 | 60 (50, 75) | 55 (40, 74) | 5 (0 to 10) | 0.196 |
| Day 2 | 70 (60, 80) | 73 (50, 85) | 0 (-10 to 5) | 0.598 |
| Day 3 | 70 (60, 85) [3] | 70 (60, 80) [1] | 0 (-5 to 5) | 0.847 |
| Day 4 | 70 (60, 80) [7] | 75 (60, 85) [6] | 0 (-10 to 5) | 0.453 |
| Day 5 | 70 (60, 80) [23] | 75 (65, 80) [20] | 0 (-10 to 5) | 0.610 |
| Noise (point) |  |  |  |  |
| Day 1 | 80 (70, 80) | 80 (70, 80) | 0 (0 to 10) | 0.226 |
| Day 2 | 80 (80, 80) | 80 (73, 80) | 0 (0 to 0) | 0.773 |
| Day 3 | 70 (70, 85) [3] | 80 (70, 90) [1] | 0 (0 to 0) | 0.518 |
| Day 4 | 80 (70, 90) [7] | 80 (70, 90) [6] | 0 (0 to 0) | 0.333 |
| Day 5 | 80 (70, 80) [23] | 80 (70, 80) [20] | 0 (0 to 0) | 0.691 |

Data are median (interquartile range). *P* values in bold indicate <0.05.

^a^ Richards-Campbell Sleep Questionnaire is a 5-item questionnaire. Responses are recorded on a 100-millimeter visual-analogue scale, with higher scores representing better sleep and the mean of these five items representing the overall score (primary measure). The Richards-Campbell Sleep Questionnaire also included a sixth item, not included in the overall score, evaluating perceived nighttime noise (visual-analogue scale range: 0 for “very noisy” to 100 for “very quiet”).

^b^ Calculated as dexmedetomidine group minus placebo group.

**Supplement Table S3.** Postoperative results of pain intensity.^a^

|  | **Dexmedetomidine (n=76)** | **Placebo (n=76)** | **Median difference (95% CI) ^b^** | ***P* value** |
| --- | --- | --- | --- | --- |
| NRS of pain, at rest (point) |  |  |  |  |
| Day 1-am | 1 (0, 2) | 2 (0, 3) | 0 (-1, 0) | 0.051 |
| Day 1-pm | 1 (0, 2) | 2 (0, 2) | 0 (-1, 0) | **0.038** |
| Day 2-am | 1 (0, 2) | 1 (0, 2) | 0 (0, 0) | 0.541 |
| Day 2-pm | 0 (0, 2) [3] | 1 (0, 2) [1] | 0 (0, 0) | 0.524 |
| Day 3-am | 0 (0, 2) [3] | 0 (0, 2) [1] | 0 (0, 0) | 0.703 |
| Day 3-pm | 0 (0, 2) [7] | 0 (0, 2) [6] | 0 (0, 0) | 0.739 |
| Day 4-am | 0 (0, 1) [7] | 0 (0, 1) [6] | 0 (0, 0) | 0.478 |
| Day 4-pm | 0 (0, 1) [23] | 0 (0, 1) [20] | 0 (0, 0) | 0.550 |
| Day 5-am | 0 (0, 1) [23] | 0 (0, 2) [20] | 0 (0, 0) | 0.409 |
| Day 5-pm | 0 (0, 0) [37] | 0 (0, 1) [32] | 0 (0, 0) | 0.289 |
| NRS of pain, with movement (point) |  |  |  |  |
| Day 1-am | 3 (3, 4) | 4 (3, 5) | 0 (-1, 0) | 0.083 |
| Day 1-pm | 3 (2, 4) | 4 (3, 4) | 0 (-1, 0) | 0.119 |
| Day 2-am | 3 (2, 4) | 3 (2, 4) | 0 (0, 0) | 0.740 |
| Day 2-pm | 3 (2, 3) [3] | 3 (2, 4) [1] | 0 (0, 0) | 0.831 |
| Day 3-am | 3 (2, 3) [3] | 3 (2, 4) [1] | 0 (-1, 0) | 0.647 |
| Day 3-pm | 2 (2, 3) [7] | 3 (2, 4) [6] | 0 (-1, 0) | 0.132 |
| Day 4-am | 2 (2, 3) [7] | 2 (2, 3) [6] | 0 (-1, 0) | 0.364 |
| Day 4-pm | 2 (1, 3) [23] | 2 (2, 3) [20] | 0 (-1, 0) | 0.711 |
| Day 5-am | 2 (1, 3) [23] | 2 (1, 2) [20] | 0 (-1, 0) | 0.301 |
| Day 5-pm | 2 (1, 3) [37] | 2 (1, 3) [32] | 0 (-1, 0) | 0.513 |

Data are median (interquartile range). Numbers in square brackets indicate patients with missing data due to hospital discharge. *P* values in bold indicate <0.05.

^a^ Postoperative pain intensity is assessed with Numeric Rating Scale (NRS; an 11-point scale where 0 indicates no pain and 10 indicates the worst pain).

^b^ Calculated as dexmedetomidine group minus placebo group.

**Supplement Table S4.** Postoperative results of Richmond Agitation-Sedation Scale (point).^a^

|  | **Dexmedetomidine (n=76)** | **Placebo (n=76)** | **Median difference (95% CI) ^b^** | ***P* value** |
| --- | --- | --- | --- | --- |
| Day 1-am | 0 (0, 0) | 0 (0, 0) | 0 (0, 0) | 0.993 |
| Day 1-pm | 0 (0, 0) | 0 (0, 0) | 0 (0, 0) | 0.317 |
| Day 2-am | 0 (0, 0) | 0 (0, 0) | 0 (0, 0) | 0.156 |
| Day 2-pm | 0 (0, 0) [3] | 0 (0, 0) [1] | 0 (0, 0) | >0.999 |
| Day 3-am | 0 (0, 0) [3] | 0 (0, 0) [1] | 0 (0, 0) | 0.324 |
| Day 3-pm | 0 (0, 0) [7] | 0 (0, 0) [6] | 0 (0, 0) | >0.999 |
| Day 4-am | 0 (0, 0) [7] | 0 (0, 0) [6] | 0 (0, 0) | 0.314 |
| Day 4-pm | 0 (0, 0) [23] | 0 (0, 0) [20] | 0 (0, 0) | >0.999 |
| Day 5-am | 0 (0, 0) [23] | 0 (0, 0) [20] | 0 (0, 0) | 0.304 |
| Day 5-pm | 0 (0, 0) [37] | 0 (0, 0) [32] | 0 (0, 0) | >0.999 |

Data are median (interquartile range). Numbers in square brackets indicate patients with missing data due to hospital discharge.

^a^ Sedation level is assessed with the Richmond Agitation Sedation Scale (RASS), with scores ranging from -5 (unarousable) to +4 (combative), and 0 indicates alert and calm.

^b^ Calculated as dexmedetomidine group minus placebo group.
